# Atypical Mucocutaneous Leishmaniasis Presentation Mimicking Rectal Cancer

**DOI:** 10.1155/2023/2768626

**Published:** 2023-04-15

**Authors:** Helina Fikre, Ermias Teklehaimanot, Rezika Mohammed, Miklol Mengistu, Bewketu Abebe, Johan van Griensven, Saskia van Henten

**Affiliations:** ^1^University of Gondar, Leishmaniasis Research and Treatment Center, Gondar, Ethiopia; ^2^University of Gondar, Department of Pathology, Gondar, Ethiopia; ^3^University of Gondar, Department of Surgery, Gondar, Ethiopia; ^4^Institute of Tropical Medicine, Antwerp, Belgium

## Abstract

Cutaneous leishmaniasis is a neglected tropical disease affecting mostly the exposed skin, causing severe and disfiguring lesions in Ethiopia. In this report, we present two cases of atypical mucocutaneous leishmaniasis; one HIV positive and one HIV negative patient. *Cases*. A 32-year-old male HIV patient presented with 40 days of bleeding per-rectum and a perianal lesion of 5 years. An erythematous nontender plaque measuring 5 cm by 5 cm was observed over the right perianal area with circumferential constricting firm swelling of the rectum. The patient was cured with AmBisome and miltefosine after an incisional biopsy revealed leishmaniasis. A 40-year-old presented with bleeding per-rectum and stool incontinence of 3 months, generalized body swelling of 2 months, and mass around his anus for ten years. A 6 by 3 cm indurated ulcerating mass surrounding the anus and a fungating circumferential mass of 8 cm were seen above the proximal anal verge. An excisional biopsy revealed leishmaniasis, and the patient was treated with AmBisome but passed away due to complications with colostomy diarrhea. *Conclusion*. Clinicians should consider atypical mucocutaneous leishmaniasis as a possible diagnosis in patients with chronic skin lesions resembling hemorrhoids and colorectal masses, especially in endemic areas such as Ethiopia, regardless of their HIV status.

## 1. Background

Infections caused by *Leishmania* represent a wide range of diseases, including *cutaneous leishmaniasis* (CL) and *visceral leishmaniasis* (VL). In total, 1.5 million cases of CL are registered annually, with Ethiopia among the countries with a high burden of CL with an estimation of 20,000 to 30,000 cases per year [1].

*Leishmania aethiopica* is the main cause of CL in Ethiopia causing severe forms of CL which do not respond well to most treatments [2]. Three clinical presentations of CL have been reported in the country including localized CL (LCL) which is characterized by localized papular or nodular lesions at the site of the sand-fly bite. The LCL lesion is usually painless, pink, and round with well-defined raised edges, and in some cases, it could be self-limiting. Also reported in the country is mucocutaneous leishmaniasis (MCL) characterized by mucosal (nasal, oral, and rarely on pharynx and larynx) involvement either by direct bites on the mucosal surface or by extension of the localized CL (1,2). Diffuse CL (DCL) is the third type, and is characterized with multiple skin lesions which are nonulcerating, papular, nodular, and plaque features involving most parts of the body [2].

Although an atypical presentation of VL involving intestinal HIV/VL coinfection has been described in Ethiopia [3], to our knowledge, there are no other available cases reported on MCL involving the intestine. Here, we report on the atypical presentation of MCL with involvement of the anus and the rectum, mimicking rectal cancer.

## 2. Case Presentations

### 2.1. Case 1

In Oct 2017, a 32-year-old male presented with intermittent bleeding per-rectum after defecation for 40 days with associated cough and fever. The patient had noticed a mass over the anus for five years but did not seek treatment as the lesion was not painful. He had visited the surgical unit at Gondar University Hospital, and he was under investigation for possible colorectal carcinoma.

At the presentation, he was well-looking, with normal vital signs. On abdominal examination, he had a spleen palpable 5 cm below the left costal margin, a liver palpable 2 cm below the right costal margin, and a 5 cm by 5 cm erythematous nontender plaque over the inner right buttock, perianal area. Per-rectum examination showed a circumferential firm rectal swelling 4 cm from the anal verge.

The abdominal CT scan showed a circumferential rectal mass 2.5 cm thick with extension to the mesorectum and thickening of the mesorectal facia (suggestive of rectal cancer with rectal and mesorectal extension). It also showed mesorectal lymphadenopathy (stage III) and hepatosplenomegaly. The conclusion of the CT scan was rectal cancer plus hepatosplenomegaly. Chest X-ray was normal.

An endoscopic biopsy from the rectum showed three fragments with unremarkable mucosa except for focal ulceration. The submucosa showed intense lymphocytic inflammation, and numerous macrophages were stuffed with intracellular *Leishmania amastigotes*. No feature of malignancy was seen in the biopsy. Fine needle aspiration (FNA) from the perianal lesion showed Leishman Donovan bodies (grade+3 amastigotes). Splenic aspiration was carried out to rule out concomitant VL but was negative for *Leishmania amastigotes*. An HIV test was carried out at admission, and he was found to be seropositive with a CD4 count of 306.

The patient was treated with (liposomal amphotericin B) AmBisome 5 mg/kg for 8 doses initially after which rectal bleeding stopped, and his fever recovered. On physical examination, he had a perianal 4 cm by 2 cm mass with central ulceration, which had decreased in size but still showed an active CL lesion. Therefore, treatment was extended with AmBisome 5 mg/kg for 8 doses plus miltefosine 100 mg for 28 days (2 × 50 mg daily). Antiretroviral therapy was planned to be initiated after counseling.

After treatment completion, the perianal mass flattened to 3 by 2 cm, with no significant rectal mass appreciated on physical examination. Fine needle aspiration from the perianal healed lesion was negative for Leishman Donovan bodies. A repeated abdominal CT scan showed a circumferential rectal mass of 1 cm and splenomegaly, indicating a reduction in the rectal lesion size. The patient was followed for 2 years, without any recurrence.

### 2.2. Case 2

In January 2019, a 40-year-old male patient presented with bleeding per-rectum, fever, loss of appetite, weight loss, and stool incontinence of 7 months, associated with generalized body swelling of 2 months. He had a mass around his anus for 10 years, which he considered as hemorrhoids and was not treated medically. For this, he visited Gondar University Hospital and was admitted to the medical unit for 2 weeks. His diagnosis was rectal carcinoma with anal incontinence, and he was transferred to the surgical unit, and he was appointed for further diagnostic workup. However, he came later than the appointment he was given, and after his symptoms had worsened. On investigation, an abdominal CT scan showed a 4 cm long polypoid enhancing mass in the anorectal junction with adjacent nodular fat stranding in the mesorectum. There was minimal ascites, with bilateral pleural effusion. The conclusion was an anorectal mass, likely carcinoma, with ascites and bilateral pleural effusion. Serum albumin was 1.2 mg/dl, and an HIV test was negative.

With the suspicion of rectal carcinoma, a diverting loop sigmoid colostomy was carried out through a left lower quadrant incision, and an incisional biopsy was performed. Multiple samples were taken from the rectal mass, and a wedge resection biopsy from the anal ulceration was performed. A colostomy bag was left in situ, and the patient was admitted to the surgical ward.

While awaiting diagnosis, the patient developed hospital-acquired pneumonia and was initiated on intravenous ceftriaxone, intravenous metronidazole, and intravenous tramadol at the surgical ward.

**Figure 1:**
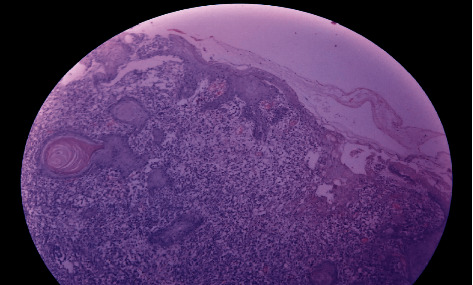
Low power microscopy of the histologic section from the perianal lesion showing skin-covered tissue with a focus on ulceration and intense subepithelial mixed inflammatory cell infiltrates (H&E), case 2.

**Figure 2:**
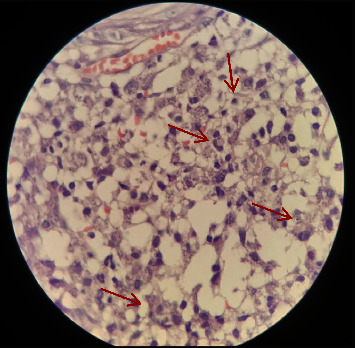
High power microscopy showing numerous foamy macrophages stuffed with an intracellular parasite (arrow) along with other inflammatory cells (H&E) case 2.

On the 24^th^ day postsurgery, his biopsy result arrived. Microscopic examination from both the anal ulcer and rectal mass showed lymphoplasmacytic cell infiltrates along with foamy macrophages stuffed with intracellular *Leishmania* amastigotes (Figures 1 and 2). No features of malignancy were seen in both tissue sections. Other organisms mimicking *Leishmania amastigotes* such as *Histoplasma* were excluded morphologically on histology. Therefore, a diagnosis of leishmaniasis was made, and the patient was transferred to the Leishmaniasis Research and Treatment Center (LRTC).

The photographic image of histopathology was not available for case 1.

Once leishmaniasis was confirmed, the patient was transferred from the surgical ward to LRTC. At presentation to LRTC, his general appearance was chronically sick-looking and emaciated. His blood pressure was low at 80/50 mmHg, and his weight was 42 kg; height was not measured as the patient could not stand; other vital signs were in the normal range. On abdominal examination, there was a fluid collection but no organomegaly, and a colostomy bag was present on the left lower quadrant of the abdomen.

Per-rectum examination showed a 6 by 3 cm indurated ulcerating mass surrounding the anus and a fungating circumferential rectal mass 8 cm above the proximal anal verge, with loss of anal tone.

He was started on AmBisome 5 mg/kg IV daily and once weekly for 8 doses. While hospitalized, he developed gastritis with intractable vomiting, after which he developed severe hypokalemia with a serum potassium level of 2.2 mmol/L and elevated creatinine level of 1.7 mg/dl. For this, he was started on potassium (KCL) IV infusion, IV omeprazole, and IV hydration. Though the perianal lesion was decreasing to 4 by 3 cm, his general condition worsened due to hypoalbuminemia and massive uncontrollable colostomy diarrhea, and the patient died 18 days after the leishmaniasis diagnosis.

## 3. Discussion

There have been case reports in Ethiopia and Mexico regarding intestinal involvement in leishmaniasis, but they were in relation to VL and HIV coinfection [3, 4]. In addition, there are few case reports of VL with intestinal involvement in India and Italy which occurred in immunocompetent individuals [5, 6]. In our report the possibility of visceral involvement was ruled out by splenic aspiration in the first case, and the second case had no splenomegaly.

Both cases had prior skin lesions around the anus which over the years involved the rectal mucosa, after which both patients developed diarrhea and bleeding per-rectum. Samples from skin lesions and rectal mucosa in both cases showed *Leishmania* amastigotes which supports the diagnosis of atypical MCL. Unfortunately, the species could not be identified by molecular techniques due to unfavorable storage conditions of the samples. Therefore, we classified both cases as MCL since for both, parasitological presence of parasites was not shown in the viscera but was detected in the skin and mucosa. To our knowledge, there are no reported cases of MCL mimicking rectal cancer.

Histopathologically, *Leishmania* organisms are easily visualized on routine hematoxylin and eosin sections, and numerous amastigotes typically distend histiocytes [7, 8]. In both of our cases, subepithelial lymphoplasmacytic cells infiltrate along with foamy macrophages stuffed with intracellular microorganisms morphologically in-confirming *Leishmania* amastigotes.

One differential for histopathological findings of CL is histoplasmosis; organisms are of a similar size to *Leishmania* but stain readily with silver stains, show narrow-based budding, and have a periorganism halo. The late granulomatous stages of leishmaniasis may resemble other granulomatous diseases such as sarcoidosis [8]. In our case, histoplasmosis was ruled out morphologically as the *Leishmania* parasite was readily identifiable with classic morphology. Sarcoidosis was easily ruled out as we have little granuloma formation and numerous parasites.

Few clinical differential diagnoses of LCL, MCL, and DCL are known including eczema, leprosy, syphilis, and fungal infection. For MCL, the top differentials include tuberculosis, histoplasmosis, sarcoidosis, and skin cancer [1, 8]. However, rectal cancer was not listed so far. In our patients, the first diagnosis considered was rectal cancer; since there was no initial clinical suspicion of MCL, patients had to pass through invasive surgical diagnostic procedures. In the second case, the patient had loop colostomy which led to colostomy diarrhea and related complications that probably had a role in his death.

There is no standardized treatment for MCL as per Ethiopian treatment and diagnosis guidelines [1]. Sodium stibogluconate (SSG), AmBisome, miltefosine, and paromomycin are also recommended as systemic treatment. For VL, AmBisome is given to patients who have contraindications for SSG, such as being severely malnourished or immunocompromised. Miltefosine is not always available, but it is a recommended treatment for HIV/VL coinfected patients in combination with AmBisome [1, 9]. Treatment duration varies depending on patients' clinical and parasitological responses. In our case, we used Ambisome followed by AmBisome and miltefosine for the first patient as he was HIV positive. Although the second patient was seronegative, he was still started on AmBisome as he was severely malnourished.

In this report, we emphasize lack of suspicion and late diagnosis of the MCL patient with rectal lesions, which leads to invasive surgical procedures. This is illustrated by our second case who had to go under loop colostomy which may have increased his morbidity and contributed to his death.

## 4. Conclusion

The current evidence suggests that atypical presentation of MCL can occur in patients irrespective of their HIV status. Therefore, we urge clinicians to consider CL in endemic areas when faced with chronic skin lesions presenting as hemorrhoid and rectal masses.

## Data Availability

Data sharing does not apply to this article as no new data were created or analyzed in this study.

## Abbreviations

CL: Cutaneous leishmaniasis
MCL: Mucocutaneous leishmaniasis
HIV: Human immune virus
LRTC: Leishmaniasis Research and Treatment Center.
